# Efficacy and safety of acupuncture for hand osteoarthritis: study protocol for a multi-center, randomized, sham-controlled clinical trial

**DOI:** 10.1186/s13018-023-03570-6

**Published:** 2023-02-06

**Authors:** Weiming Wang, Hangyu Shi, Yan Liu, Yuanjie Sun, Yu Chen, Zhishun Liu

**Affiliations:** 1 Department of Acupuncture and Moxibustion, Guang’anmen Hospital, China Academy of Chinese Medical Sciences, Beijing, China; 2 Beijing University of Chinese Medicine, Beijing, China; 3 Key Laboratory of Chinese Internal Medicine of Ministry of Education, Dongzhimen Hospital, Beijing University of Chinese Medicine, Beijing, China; 4 New Zealand College of Chinese Medicine, Auckland, New Zealand

**Keywords:** Acupuncture, Hand osteoarthritis, Clinical trial, Efficacy and safety

## Abstract

**Background:**

Hand osteoarthritis (OA) is a prevalent disorder in the general population. Patients with hand OA often report symptoms of pain, stiffness, and functional limitations, which cause clinical burden and impact on quality of daily life. However, the efficacy of current therapies for hand OA is limited. Other therapies with better effects and less adverse events are in urgent need. Acupuncture is well known for analgesia and has been proved effective in treating basal thumb joint arthritis. This study aims to clarify the efficacy and safety of acupuncture treatment for clinical symptomatic improvement of hand OA.

**Methods:**

This will be a sham-controlled, randomized, multi-center clinical trial. A total of 340 participants will be recruited and randomly allocated to either traditional acupuncture group or sham acupuncture group. All participants will receive 12 treatment sessions over 4 weeks and 2 follow-up assessments in the following 3 months at week 8 and week 16. The primary outcome will be the proportion of responders at week 5. Secondary outcomes will include visual analog scale, Australian Canadian Osteoarthritis Hand Index, Functional Index for hand OA, the number of symptomatic joints, hand grip strength and pinch strength, global assessment, the World Health Organization Quality of Life abbreviated version and expectations. Safety will be evaluated during the whole process of the trial. All outcomes will be analyzed following the intention-to-treat principle.

**Discussion:**

This prospective trial will provide high-quality evidence on evaluating the efficacy and safety of acupuncture treatment for hand OA. Results of this trial might contribute in offering a new option to clinical recommendations.

*Trial registration* ClinicalTrials.gov Identifier: NCT05267093. Registered 23 February 2022.

## Introduction

Hand osteoarthritis (OA) is a common disorder causing disabling pain, stiffness, reduced grip and/or pinch force and loss of motion [1–3]. The overall prevalence of hand OA was estimated to be 8.99–12% around the world [3–5]. Symptomatic hand OA is more frequently seen in the elder population, and the prevalence in women is twice higher than in men [6, 7]. Radiographic features such as joint space narrowing, osteophyte formation, and subchondral sclerosis are usually identified by plain radiography [2]. Hand OA can affect single or multiple joints, and the dominant hand is more likely to be affected due to over usage [8]. Risk factors consist of age, joint hypermobility, trauma, inflammation and gene locus related to cartilage calcification, which contribute complicatedly to the etiology and aid the change of joint structure [1, 2, 8–10]. Therefore, hand OA is a heterogeneous disorder that is hard to predict.

At present, no disease-modifying treatment is available to cure hand OA by reducing pain and improving joint motion [11]. Out of safety reasons, the first-line choice for hand OA treatment is topical pharmacologic intervention rather than oral medicine [11, 12]. It is reported that topical non-steroidal anti-inflammatory drugs (NSAIDs) have significant positive effect for hand OA after 4-week treatment [13]. However, the long-term effect is not clear [11, 14], and the difference between topical pharmacologic intervention and placebo are still in argument [15]. Although considered much safer than systematic drugs, the topical drugs require additional caution in use because it may result in skin adverse effects (AEs) with prevalence of 10–39% [16, 17]. Other available therapies, such as intra-articular steroids, oral chondroitin sulfate and dietary supplements, fail to provide certain benefit to patients with hand OA. Thus, alternative therapies with better effect and less AEs are considerably in need.

Acupuncture is an ancient non-pharmacologic therapy derived from traditional Chinese medicine and is well known for analgesia. Acupuncture has been proved an effective therapy for knee OA [18, 19], whereas, evidence on its efficacy for hand OA is extremely limited. Symptoms of hand OA are similar to those of knee osteoarthritis, including pain, loss of function and change in joint structure. Also, both knee and hand OA share similar pathophysiology of degradation of the cartilaginous matrix [1, 20]. Recent studies reported that acupuncture alleviates pain in knee OA by regulating inflammation and inhibiting hypertrophic differentiation [21], which has proved the efficacy of acupuncture treatment to osteoarthritis. Hence, acupuncture may be a promising alternative therapy for hand OA. Barnard et al. [22] reported a randomized controlled trial with 70 basal thumb joint arthritis patients comparing acupuncture and sham acupuncture (35/35). Both groups showed significant post-treatment improvement in pain relief, although no difference between acupuncture and sham acupuncture was demonstrated, and clinical evidence on follow-ups is absent.

Our study will be a 4-month multi-center, randomized, sham-controlled clinical trial designed to evaluate the efficacy and safety of acupuncture for clinical symptomatic improvement of hand OA.

## Methods

### Study design

**Fig. 1:**
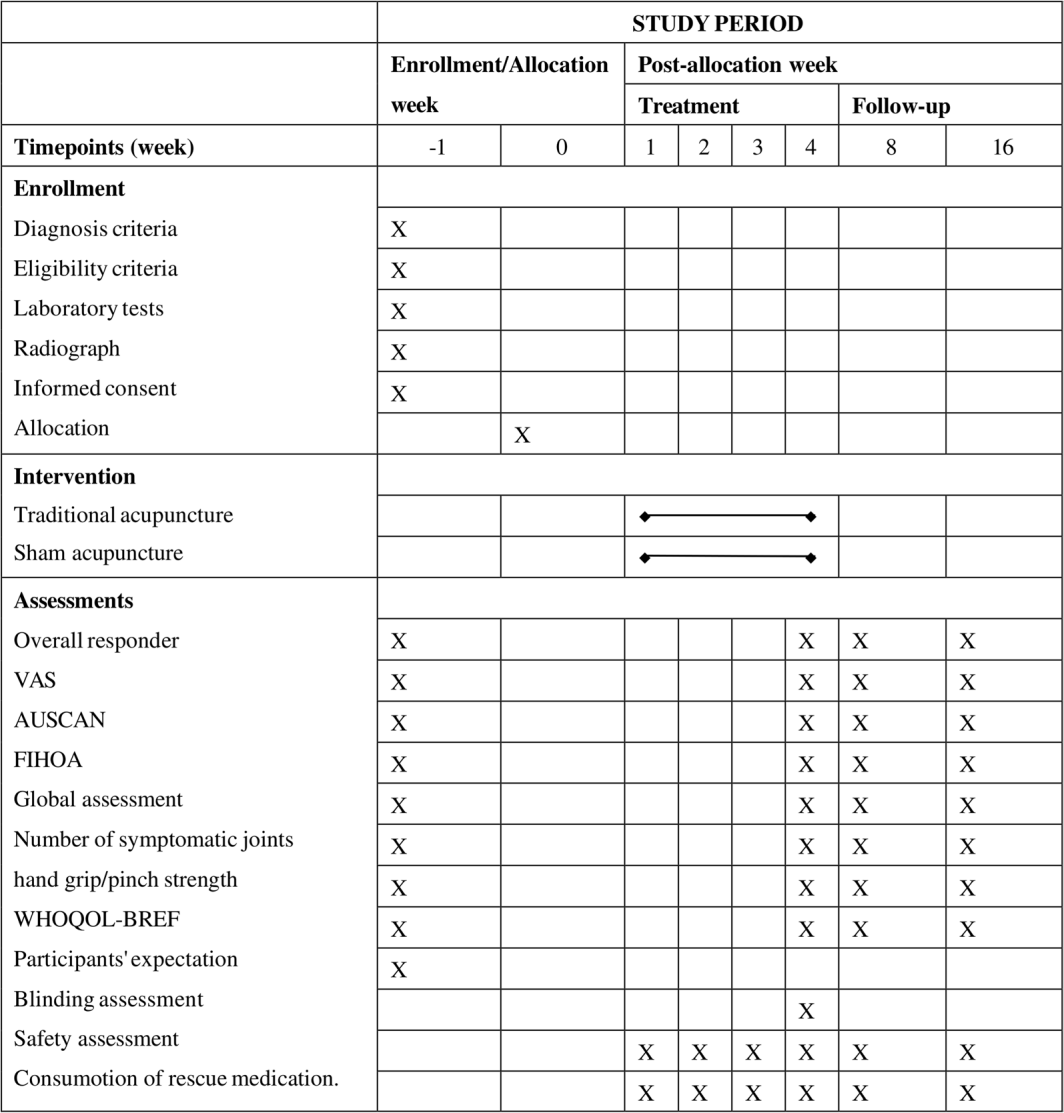
Schedule of enrolment, interventions, and assessments. VAS: Visual analog scale; AUSCAN: Australian Canadian Osteoarthritis Hand Index; FIHOA: Functional Index for hand OA; WHOQOL-BREF: World Health Organization Quality of Life abbreviated version

This multi-center, randomized, sham-controlled, participant-blinded and assessor-blinded trial will be carried out in six hospitals across Beijing, China. Patients with hand OA will be enrolled from April 2022 to October 2023. This clinical trial consists of a 4-week treatment period and a 3-month follow-up period. See study flowchart shown in Fig. 1.

This trial will be conducted in accordance with the principles of the Declaration of Helsinki. The protocol has been approved by the Institutional Review Board of Guang’anmen Hospital, China Academy of Chinese Medical Sciences (ethical approval number: 2021-131-KY) on December 15, 2021. We developed this protocol conforming to the Standard Protocol Items: Recommendations for Interventional Trials (SPIRIT) checklist and the Standards for Reporting Interventions in Controlled Trials of Acupuncture (STRITCA). The registration number at www.clinicaltrials.gov is NCT05267093.

### Randomization and allocation

Eligible participants will be randomly assigned in a 1:1 ratio to either the traditional acupuncture group (TA) or the sham acupuncture group (SA). The randomization scheme will be generated by an independent institute, Lnkmed Tech Co. Ltd. (Beijing, China), and the allocation will be performed and concealed through the Interactive Web Response System (IWRS). Stratified permuted block randomization with center-based stratification and variable block size will be applied by Lnkmed using the SAS version 9.4. IWRS will assign a code to each enrolled participant, which will be concealed from participants, outcome assessors and the statisticians. The acupuncturist will be the only one who have access to both the code and the treatment labeled with the code.

### Blinding

In this study, the participants, outcome assessors, and the statisticians will be blinded to the group allocation. The acupuncturists will not be blinded due to the characteristics of acupuncture. Sham acupuncture treatment will mimic the similar sensation of traditional acupuncture treatment using blunt-tip needles. To ensure the success of blinding, the acupuncturists will be discouraged from discussing the treatments or expected results with participants. A blinding assessment will be taken right after the participant’s last session that asks the participant to guess which treatment he/she has received.

### Recruitment

This study plans to recruit 340 patients with hand OA from six hospitals across Beijing, China. Eligible patients who meet the American College of Rheumatology (ACR) diagnosis criteria of hand OA [23] in the dominant hand will be recruited via WeChat, websites, posters and bulletin boards in the hospitals and nearby communities. The rheumatologist of each center will be in charge of the diagnosis of participants. Informed consent will be adequately explained to all eligible participants. Participants will be asked to sign the consent form before screening, all of them have the right to withdraw their consent at any time and we guarantee the confidentiality of all participants.

### Inclusion and exclusion criteria

Participants who are diagnosed as hand OA in the dominant hand according to the ACR diagnosis criteria of hand OA [23] will be considered, specifically, hand pain or stiffness in the dominant hand with at least 3 of the following features: hard tissue enlargement of at least 2 of the 10 selected joints (the 2nd and 3rd distal interphalangeal (DIP) and proximal interphalangeal (PIP) joint and the first carpometacarpal (CMC) joint of both hands); hard tissue enlargement of at least two DIP joints; swelling of fewer than three metacarpophalangeal joints; deformity of at least one of the 10 aforementioned selected joints.

Participants who meet all of the following criteria might be included: (1) diagnosed as hand OA in the dominant hand; (2) history of hand OA for at least 3 months before enrollment and history of taking NSAIDs to treat hand OA; (3) aged 18–80 years; (4) scoring at least 40 mm in visual analog scale (VAS) for the average pain intensity over the last 48 h of the dominant hand, or an increase of ≥ 20 mm in VAS score after 1-week washout for participants applying NSAIDs at the enrollment; (5) Kellgren–Lawrence grade 1, 2, or 3 in symptomatic joints on posterior-anterior radiographs; (6) negative results in both rheumatoid factor and anticyclonic citrullinated peptide; (7) able to comply with the study protocol and understand the medical information forms; (8) voluntarily sign the informed consent.

Participants who meet any of the following criteria will be excluded: (1) history or current evidence of secondary OA, or symptomatic OA of other joints requiring treatment, or any upper limb pain that may interfere with the evaluation of hand pain; (2) history of inflammatory arthritis (such as rheumatoid arthritis and psoriatic arthritis), hemochromatosis, metabolic or neuropathic arthropathies; (3) history of trauma, dislocation or operation to the hand or arm within the previous 3 months; (4) hand pain and stiffness due to tissue scarring or tendinitis; (5) skin damage or serious skin disorder of the hands; (6) in the administration of antidepressants, anticonvulsants, vascular or narcotics within the previous 10 days; (7) oral, intramuscular, intra-articular or intravenous corticosteroids, or hyaluronic acid injection within 3 months; (8) uncontrolled dangerous conditions such as cancer, uncontrolled cardiovascular disorder, hepatic/renal insufficiency or coagulation disorder; (9) afraid of needles or received acupuncture treatments within 4 weeks.

### Interventions

#### TA group

In each center, treatments will be performed by a licensed acupuncturist who have at least 2 years of clinical experience. Before the study starts, the acupuncturists will be extensively trained on the location, depth and angle of acupuncture.

**Fig. 2:**
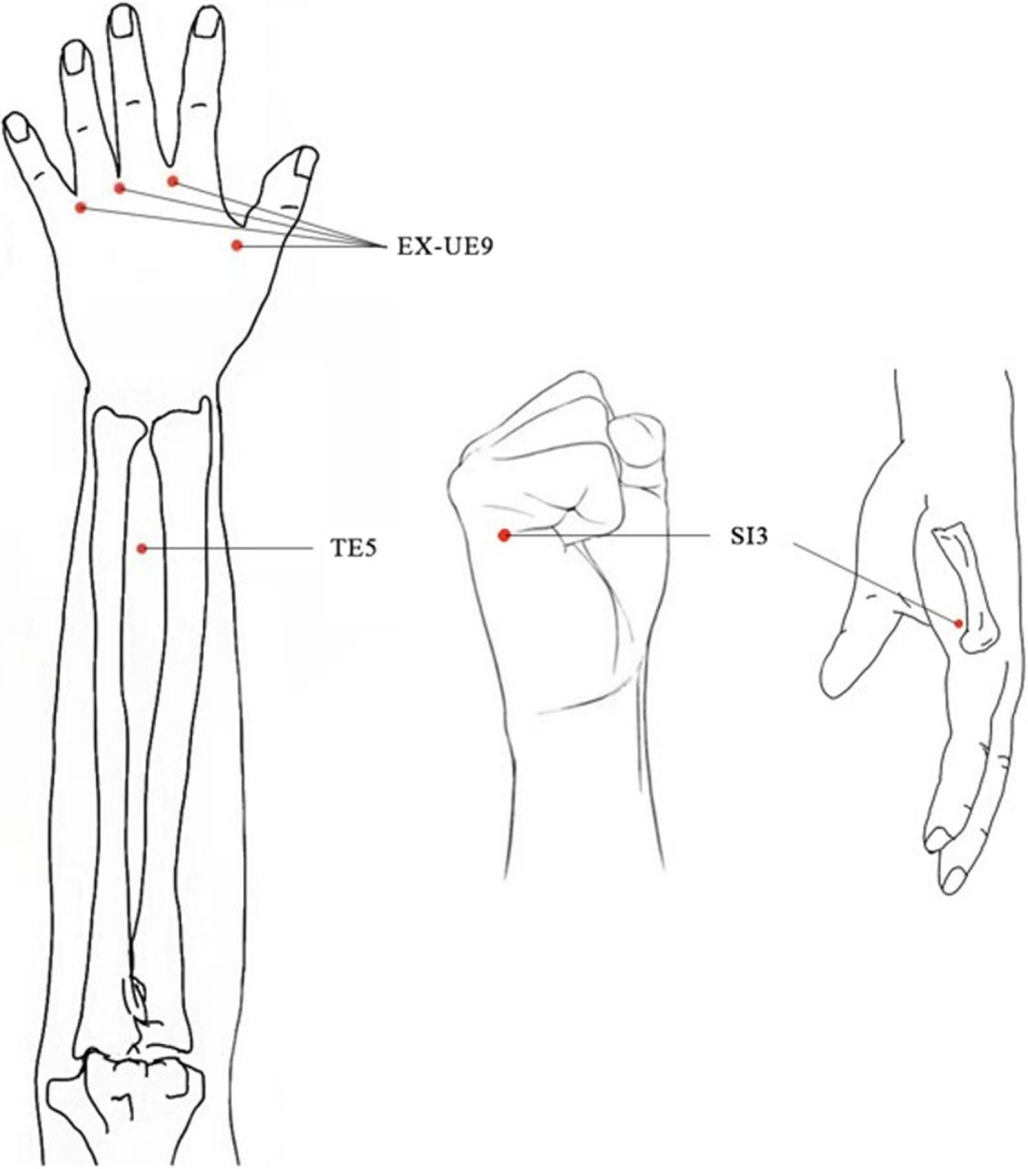
Locations of acupoints. EX-UE9: Baxie; TE5: Waiguan; SI3: Houxi

**Fig. 3:**
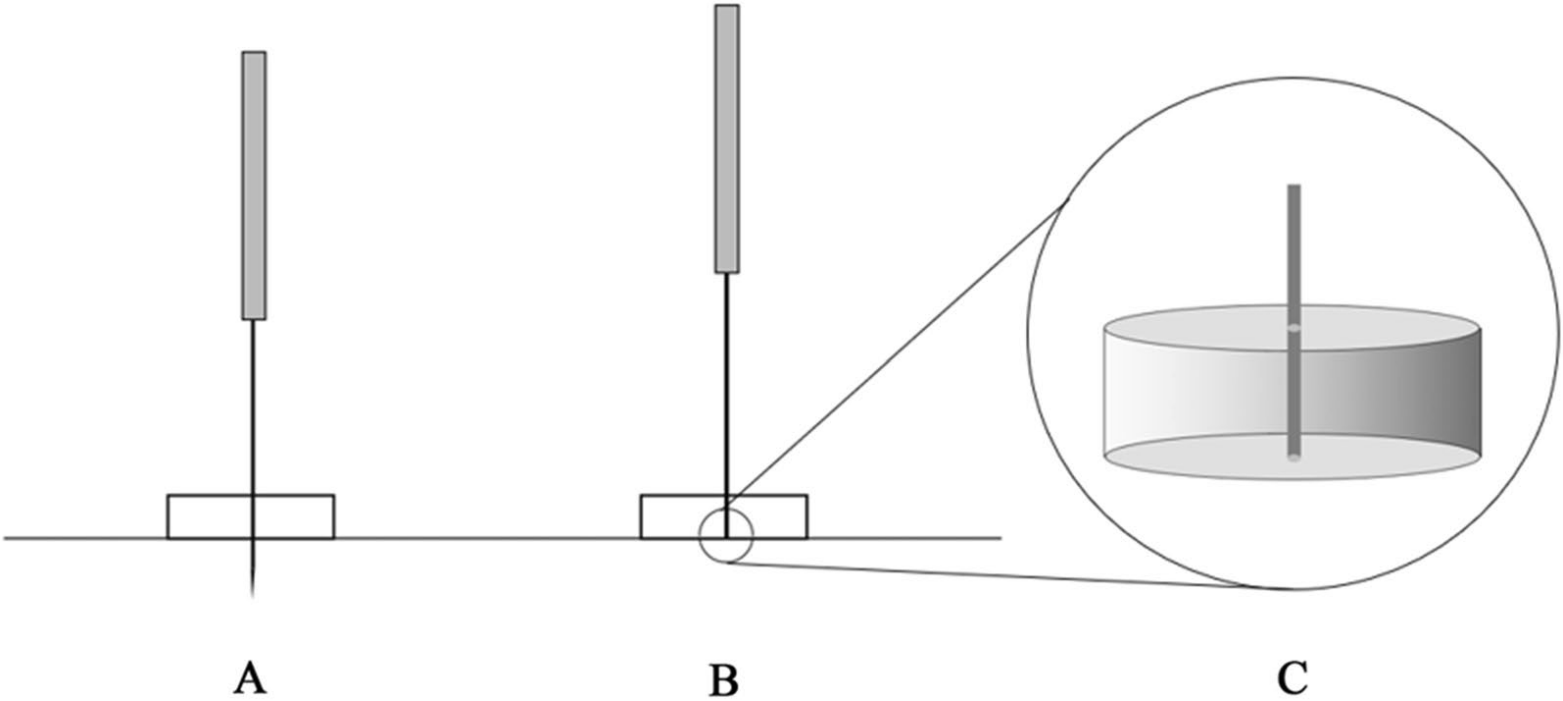
Illustration of acupuncture. **A**. traditional acupuncture; **B**. sham acupuncture; **C**. blunt tip

Acupoints are selected in accordance with the meridian theory of traditional Chinese medicine and the consensus of senior doctors, including bilateral Baxie (EX-UE9), bilateral Houxi (SI3), bilateral Waiguan (TE5) and Ashi points. All acupoints will be localized based on the World Health Organization (WHO) Standard Acupuncture Locations as exhibited in Table 1 and Fig. 2. The acupuncturist will first sterilize local skin with 75% alcohol cotton pellets and tape foam pads (Fig. 3), then insert sterile disposable needles (size 0.3 mm × 25 mm) into the acupoints and gently rotate and lift several times to obtain Qi (deqi) response. The depths and angles of needle insertion are shown in Table 1. Every treatment session will last for 30 min, and then, the needles will be gently removed by acupuncturists. Participants will receive three treatment sessions per week (every other day) for 4 consecutive weeks. Using a treatment diary, the treatment sessions will be monitored and assessed.

**Table 1:** Locations and manipulations of acupoints

	Location	Depth	Anatomy	Deqi
Baxie (EX-UE9)	Proximal to the web margins between the five fingers at the junction of the red and white skin, 4 points on each hand, 8 in total	0.2–0.5 cun	Interossei muscles	Yes
Waiguan (TE5)	On the posterior aspect of the forearm, 2 cun^a^ proximal to the dorsal wrist crease, midpoint of the interosseous space between the radius and the ulna	0.5–0.8 cun	Between the extensor digitorum and the extensor hallucis longus	Yes
Houxi (SI3)	In the depression proximal to the ulnar side of the fifth metacarpophalangeal joint, at the junction of the red and white flesh	0.2–0.5 cun	Beside the origin of the abductor digiti minimi muscle of hand	Yes
Ashi points	At the midpoint of the dorsum of the most painful joint	0.1 cun	Subcutaneous	No

^a^From the cubital crease to the wrist crease: 12 cun

#### SA group

Sham acupuncture will be performed in SA group. The specially designed blunt-tip sham needles (size 0.3 mm × 25 mm) will be utilized to mimic the sensation of TA and confuse the participants to the most extent. Acupoints used in the SA group are the same as those in the TA group. After local skin sterilized, small foam pads will be taped on acupoints to cover up the manipulation of SA. The acupuncturist will insert blunt-tip needles through foam pads (Fig. 3), leave the needles on the surface of skin without penetration, then thrust and rotate the needles to produce the sensation of piercing. Blunt-tip sham needles will remain 30 min on skin, then gently removed. The treatment sessions and frequency will be the same as in TA group. The treatment diary will also be used in SA group.

### Rescue medication

Any other therapy will not be allowed during this trial, including oral medications (such as glucosamine sulfate, corticosteroids, and chondroitin sulfate) and topical agents (such as diclofenac sodium gel and ibuprofen gel). Acetaminophen (sustained release type, 500 mg/T) will be allowed to a maximum dose of 4 tablets daily for up to 3 days in total in case of insufferable pain. Rescue medicine should not be prescribed within 60 h before any of the assessments. Usage of rescue medication or other unexpected therapies will all be recorded in detail and properly analyzed.

### Primary outcome

The primary outcome will be the proportion of responders at week 5. The proportion of responders at week 8 and week 16 will be assessed as secondary outcome. Participants will be defined as “responders” if showing at least 1 of the 3 following criteria: (1) at least a 50% decrease and absolute change from baseline ≥ 20 in visual analog scale (VAS) score on average pain intensity; (2) at least a 50% decrease and absolute change from baseline ≥ 20 in Australian Canadian Osteoarthritis Hand Index (AUSCAN); (3) at least a 20% decrease and absolute change from baseline ≥ 10 in at least two measurements of VAS score, AUSCAN and patient global assessment of improvement. The responder criteria are summarized by the Outcome Measures in Rheumatological Clinical Trials and Osteoarthritis Research Society International (OMERACT-OARSI) [24]. Responders are acknowledged to have clinically active treatment effect [25], since this assessment uses both a relative and an absolute change in three different domains (pain, function and patient’s global assessment) instead of single measurement.

### Secondary outcomes

Secondary outcomes will include VAS score on pain intensity, AUSCAN, Functional Index for hand OA (FIHOA), the number of painful joints, tender joints and swollen joints, hand grip strength and pinch strength, global assessment, the World Health Organization Quality of Life abbreviated version (WHOQOL-BREF) and the participants' expectations for acupuncture. As shown in Table 2, the outcome measurements will be assessed at baseline, week 5, week 8, and week 16 after randomization. A responsible research assistant will be assigned by each hospital to complete all outcome assessments during this trial. The research assistants will be blinded from group allocation.

**Table 2:** Primary and Secondary Outcomes

Primary outcome	*Overall responder*	
	Showing 1 of the 3 followings: ≥ 50% decrease and absolute change ≥ 20 in VAS score on average pain intensity; ≥ 50% decrease and absolute change ≥ 20 in AUSCAN; ≥ 20% decrease and absolute change ≥ 10 in at least two measurements of VAS score on average pain intensity, AUSCAN and global assessment	Week 5
Secondary outcomes	*Overall responder*	
	Showing 1 of the 3 followings: ≥ 50% decrease and absolute change ≥ 20 in VAS score on average pain intensity; ≥ 50% decrease and absolute change ≥ 20 in AUSCAN; ≥ 20% decrease and absolute change ≥ 10 in at least two measurements of VAS score on average pain intensity, AUSCAN and global assessment	Week 8, week 16
	*Pain*	
	Change in VAS score from baseline on average pain intensity Change in VAS score from baseline on maximal pain intensity The proportion of participants with an at least 15-point decrease in VAS score from baseline on average pain intensity	Week 5, week 8, week 16
	*Physical function*	
	Change in AUSCAN total score from baseline Change in AUSCAN subscale scores including pain, stiffness and physical function from baseline Change in FIHOA from baseline	Week 5, week 8, week 16
	*Patient global assessment*	
	Change in patient global assessment of improvement from baseline	Week 5, week 8, week 16
	*Joint activity*	
	Change in the number of painful joints from baseline Change in the number of tender joints from baseline Change in the number of swollen joints from baseline	Week 5, week 8, week 16
	*Hand strength*	
	Change in hand grip strength from baseline Change in hand pinch strength from baseline	Week 5, week 8, week 16
	*Quality of life*	
	Change in WHOQOL-BREF from baseline	Week 5, week 8, week 16
	*Other outcomes*	
	Participants' expectations for acupuncture at baseline Blinding assessment right after the last session Safety assessment during the trial Proportion of participants taking rescue medication Frequency and total dosage of rescue medication	

### Visual analog scale (VAS) for pain intensity

The VAS is a validated scale presented as a 100-mm line on paper, anchored by “no pain” at 0 and “worst imaginable pain” at 100. The participants will be asked to mark the line to estimate pain intensity of their dominant hand during the last 48 h. The VAS score will be measured from 0 to the participant’s mark, with a maximum score of 100 [26]. VAS scores can be categorized into four degrees: 0 (no pain), 1–34 (mild), 35–74 (moderate) and 75–100 (severe) [27]. A 15-point decrease from baseline is required to achieve Minimal Clinically Important Difference (MCID) for patients with hand OA [28]. The change in VAS score from baseline on average pain intensity and maximum pain intensity and the proportion of participants achieved MCID on average pain intensity will be measured at baseline, week 5, week 8, and week 16.

### Australian Canadian Osteoarthritis Hand Index (AUSCAN)

The AUSCAN index is designed as a self-administered questionnaire for the evaluation of both pain and physical function in hand OA. VAS scaling is presented to the participants for the measurement of pain intensity (during 5 types of activity), stiffness (in the morning), and physical function (during 9 types of activity) [29]. By presenting as a 100-mm line, verbal descriptor “no pain/no stiffness/no difficulty” is anchored on 0 mm and “the most imaginable pain/ the most imaginable stiffness/ the most imaginable difficulty” is anchored on 100 mm for the 3 dimensions. The AUSCAN subscale scores will be calculated as the average of scores within each dimension. Higher scores indicate worse condition [30]. The AUSCAN total score will be the average of scores on the 15 items above. It is a reliable and responsive self-reported measurement that eliminates observer variation. Participant will be asked to complete the AUSCAN to indicate the condition of the dominant hand during the previous 48 h at baseline, week 5, week 8, and week 16.

### Functional index for hand OA (FIHOA)

The FIHOA is an investigator-administered questionnaire developed by Dreiser [31], containing 10 questions to assess the functional impact of hand OA. Each question is scored with a four-point Likert scale ranging from 0 to 3. The total score is the sum of all questions (ranging from 0 to 30), with a higher score indicating more severe functional impairment [31]. The FIHOA index has shown internal validity and good consistency with high sensitivity to change [32]. Its association with pain, muscle strength, and EuroQol-5 dimension (EQ-5D) has also been identified [33]. The FIHOA will be performed for the dominant hand only at baseline, week 5, week 8, and week 16.

### Patient global assessment

The participants’ assessment of their global status of the dominant hands during the last 48 h will be measured using a VAS (0 represents “worst possible”, 100 represents “best possible”) [34] at baseline, week 5, week 8, and week 16. Patient global assessment has been recommended as a core outcome domain of clinical trials in hand OA.

### Joint activity

Painful joints, tender joints, and swollen joints of the dominant hand will be counted at baseline, week 5, week 8, and week 16, which will provide information on the number and distribution of symptomatic joints and evaluate joint activity. A total of 15 joints of the dominant hand will be assessed, including the PIP, DIP, metacarpophalangeal (MCP), first interphalangeal (IPJ), and first CMC joints.

### World Health Organization Quality of Life abbreviated version (WHOQOL-BREF)

The WHOQOL-BREF is a frequently used questionnaire to evaluate quality of life. It consists of four domains comprising 26 items: physical (7 items), psychological (6 items), social (3 items), environmental (8 items), and general (2 items) [35]. Each item is scored with an ordinal scale from 1 to 5, with higher scores indicating better quality of life. The raw score of each domain will be the sum of all items and will be transformed to a 0–100 scale according to the formula reported in the WHOQOL-BREF guidelines. The translated simplified-Chinese version of WHOQOL-BREF has been shown to have validity and reliability [36]. The questionnaire will be conducted at baseline, week 5, week 8, and week 16.

### Hand strength

Grip and pinch strength of the dominant hand will be assessed at baseline, week 5, week 8, and week 16. A series of tests including grip, palmar (three-jaw chuck) pinch, key (lateral) pinch, and tip (two-point) pinch have been demonstrated highly reliable for evaluating hand strength [37]. Hand grip strength will be quantified in kilograms by measuring the amount of static force a participant can squeeze around the hand dynamometer (KYTO 2324, Kangdu Electronic Manufacture Co. Ltd, Dongguan, China). Pinch strength will be measured using a Jamar digital pinch gauge (Allegro Medical, Illinois, USA), which will be placed between the radial side of index finger and thumb, between the pulp of the thumb and the index and middle fingers, and between the tip of the thumb and the index finger, respectively, to perform 3 different pinch tests. Participants are supposed to stand with their shoulders slightly adducted and neutrally rotated, and their elbows slightly extended for each of the 4 tests [38]. The mean of three trials (with intervals of at least 30 s) for each test will be calculated and documented as final score.

### Expectations for acupuncture

At baseline, the following question will be elicited to evaluate the participants’ expectations for acupuncture: “What level of improvement do you expect from acupuncture for your hand OA?” Participants can choose one of the alternative answers including “no improvement”, “slight improvement”, “moderate improvement”, “extreme improvement”, and “unclear”.

### Safety assessment

Any acupuncture-associated adverse event (AE), such as dizziness, palpitations, local hematomas, or infection, and any other AE irrelevant to acupuncture, will be carefully observed by the acupuncturist and documented on the adverse event sheet. Serious AEs (such as fatal or life-threatening, disabling, resulting in hospitalization) will be informed to the Medical Ethics Committee of Guang’anmen Hospital within 24 h. After each session, the researchers will document AEs if happens. The trial will be immediately suspended in case of serious AEs.

### Data management and quality control

The initial data will be filled in case report forms (CRFs) by the responsible outcome assessor and immediately entered into the Electronic Data Capture (EDC) system provided by Lnkmed. The clinical research associates (CRAs) will conduct monitoring activities through the website every week to enhance the quality of trial. The CRFs will be required from the clinical sites when they accomplish all the recruitment, treatment, and assessment of participants. All data on the EDC system will be locked after confirmations are presented by two independent CRAs that data online are consistent with data in the CRFs. Any deviation from the study protocol will be reported in time.

Researchers in all clinical sites will be jointly trained by the principal investigator before we carry out the trial. Acupuncture treatments will be performed by licensed acupuncturists with at least 2 years of experience in clinical practice. Both TA and SA are safe for participants in our trial; thus, no intervention modification is permissible. Considering the management of AEs will be the same in either TA group or SA group and the trial will be suspended for all serious AEs, there will be no unblinding during the trial. Group allocation will be kept by Lnkmed and revealed after the completion of statistical analysis. Participants in the SA group will receive 12 sessions of supplementary TA treatment if they want. Withdrawals or dropouts will be clearly documented during the trial.

### Sample size

According to the unpublished preliminary data collected from 25 participants (12 in acupuncture group and 13 in sham acupuncture group) from our recent study, the response rate of acupuncture for 4 weeks was 83.3%, the response rate of sham acupuncture for 4 weeks was 69%. Calculations was performed based on two-tailed testing with a 5% alpha, a desired power set at 80%, and accounting for a 20% dropout rate after 4 months. The allocation ratio was 1, resulting in 170 participants in the TA group and 170 participants in the SA group.

### Statistics

The continuous data will be represented as means with standard deviations (SD) or medians with interquartile ranges (IQR). For normally distributed measurement data, changes in the variables from baseline will be examined by independent samples *t* test. For non-normal distribution data, nonparametric test will be used. The quantitative data will be presented as frequencies (number of cases) and relative frequencies (percentages). Categorical variables will be compared using the Chi-square (*χ*^2^) test or the Fisher exact test.

The primary outcome will be assessed in the full analysis set. The full analysis set will include all randomly assigned patients who received at least one session of treatment. Safety will be also assessed in this set. Missing data on the primary outcome will be imputed by using the multiple imputation method under the missing-at-random assumption.

All data analysis will be performed using SPSS V.20.0 (IBM SPSS Statistics; IBM Corp, Somers, NY), a two-tailed *P* value < 0.05 will be considered statistically significant.

## Discussion

The objective of this study is to evaluate the efficacy and safety of acupuncture for clinical symptomatic improvement of hand OA. Despite the fact that hand OA is a prevalent disorder around the world, the treatment is far from satisfaction. Some patients do not respond to non-pharmacological treatments, while adverse effects of pharmacological treatments cannot be ignored. Therefore, other complementary treatments are needed, but the number of clinical trials in hand OA is extremely limited. In China, acupuncture has been widely used to treat patients with hand OA, significantly reducing joint pain. The effect of acupuncture on delaying cartilage degeneration and inhibiting inflammation has been identified on rats with knee OA [39–41], which might indicate potential mechanism underlying the effect of acupuncture for hand OA. However, evidence is insufficient to support the beneficial effect of acupuncture.

This multi-center clinical trial will clarify the efficacy and safety of acupuncture for hand OA by comparing acupuncture group with sham acupuncture group with 1-month treatment and 3-month follow-up. For the purpose of increasing external validity, as well as reducing selection bias, a total of 340 participants will be recruited at 6 scattered hospitals, covering various clinics that patients with hand OA might seek medical care at. Randomization and allocation will be performed centrally by an independent institution. Group allocation will be blinded to the participants, outcome assessors, statisticians and CRAs. Our primary outcome measure will be the overall response rate, a powerful measurement proposed by the OMERACT-OARSI [24]. Other outcomes will be in consistent with the core domains suggested by consensus of the OMERACT 12 hand OA workshop [42] including pain (both absolute change and effective rate), physical function, patient global assessment, joint activity, health-related quality of life and hand strength. All researchers involved will be equally instructed by the principal investigator, and CRAs will be responsible for supervision and quality control.

The main limitations of this study are: (1) the acupuncturists will not be blinded since they will be performing treatments, which will lead to potential bias; (2) the population surveyed is not demographically diverse enough to be representative of all hand OA populations and thus generalization of the results must be considered carefully; (3) The radiological index for hand OA, which has been reported to be an important outcome that may be related to the progression of hand OA, was not selected as one of the outcomes due to funding limitations.

## Conclusion

This parallel group, randomized, sham-controlled clinical trial will investigate the efficacy and safety of acupuncture for hand OA. It is expected that this study may provide reliable data on the evidence of applying acupuncture to treat hand OA and may expand the possible range of treatment options.

## Data Availability

Data are available upon reasonable request.
